# The Spatio-Temporal Characteristics and Influencing Factors of Covid-19 Spread in Shenzhen, China—An Analysis Based on 417 Cases

**DOI:** 10.3390/ijerph17207450

**Published:** 2020-10-13

**Authors:** Shirui Liu, Yaochen Qin, Zhixiang Xie, Jingfei Zhang

**Affiliations:** 1 College of Environment and Planning, Henan University, Kaifeng 475004, China; liushirui@henu.edu.cn (S.L.); zhixiang1108@163.com (Z.X.); 18211719372@163.com (J.Z.); 2 Key Laboratory of Geospatial Technology for Middle and Low Yellow River Regions, Henan University, Kaifeng 475004, China; 3 Key Research Institute of Yellow River Civilization and Sustainable Development, Henan University, Kaifeng 475001, China

**Keywords:** COVID-19 outbreak, epidemic site, spatio-temporal characteristics, influencing factors, Shenzhen City

## Abstract

The global pandemic of COVID-19 has made it the focus of current attention. At present, the law of COVID-19 spread in cities is not clear. Cities have long been difficult areas for epidemic prevention and control because of the high population density, high mobility of people, and high frequency of contacts. This paper analyzed case information for 417 patients with COVID-19 in Shenzhen, China. The nearest neighbor index method, kernel density method, and the standard deviation ellipse method were used to analyze the spatio-temporal characteristics of the COVID-19 spread in Shenzhen. The factors influencing that spread were then explored using the multiple linear regression method. The results show that: (1) The development of COVID-19 epidemic situation in Shenzhen occurred in three stages. The patients showed significant hysteresis from the onset of symptoms to hospitalization and then to diagnosis. Prior to 27 January, there was a relatively long time interval between the onset of symptoms and hospitalization for COVID-19; the interval decreased thereafter. (2) The epidemic site (the place where the patient stays during the onset of the disease) showed an agglomeration in space. The degree of agglomeration constantly increased across the three time nodes of 31 January, 14 February, and 22 February. The epidemic sites formed a “core area” in terms of spatial distribution and spread along the “northwest–southeast” direction of the city. (3) Economic and social factors significantly impacted the spread of COVID-19, while environmental factors have not played a significant role.

## 1. Introduction

In December 2019, COVID-19 patients were identified in Wuhan, Hubei Province; the disease then spread widely within a short period. Confirmed cases were subsequently reported in countries around the world. On 11 March 2020, the World Health Organization (WHO) declared COVID-19 a “pandemic” and called on all countries to take urgent and positive action. As of 12 August 2020, more than 200 countries have reported a total of 20,433,603 cases of COVID-19, with a cumulative death toll of 741,220 [1]. The COVID-19 pandemic is a serious threat to human health and social development, causing physical damage and possible death to infected persons [2,3], affecting the living conditions and behavior patterns of the general public [4], and causing psychological panic and economic losses to society [4,5].

Highly populated areas face particularly high risks from the pandemic. Urbanization involves the concentration of populations in cities. A metropolis, with its highly developed economy and perfect infrastructure, generally has the characteristics of high population density, strong mobility of people, and high frequency of contact. The COVID-19 outbreak has made metropolises key and difficult areas for epidemic prevention and control.

COVID-19 spreads quickly and widely, showing different characteristics from other epidemics. Following the COVID-19 outbreak, the Chinese government has implemented strict control measures to try to contain the spread of the epidemic. This has included sealing off cities, suspending schools, and restricting human movement. The facts show that China has achieved good results in the prevention and control of the COVID-19 epidemic. By analyzing the epidemic situation in China, scholars have provided timely experiences and lessons to prevent and control COVID-19 around the world. For example, Li et al. (2020) analyzed the early spread of COVID-19 in Wuhan, concluding that human-to-human transmission had already occurred among close contacts, and that necessary measures were required to curb the spread of the virus [6]. Prem et al. (2020) noted that during the COVID-19 outbreak, Wuhan implemented a number of intervention measures to contain the spread of the virus, providing breathing space for medical treatment. This ultimately significantly reduced the number of infected cases [7]. Liu et al. (2020) evaluated a series of intervention strategies to prevent and control COVID-19 transmission in Henan Province, hypothesizing that appropriate intervention is the key to preventing and controlling COVID-19 [8]. Zhu et al. (2020) found a significant relationship between COVID-19 transmission and air pollution in China [9]. Research by Hao et al. (2020) found that the adoption of public health interventions reduced the cumulative number of infections in Wuhan by 96% as of 8 March [10]. Pan et al. (2020) reviewed the 92-day anti-epidemic process in Wuhan, dividing the spread of the epidemic in Wuhan into five stages [11]. Li et al. (2020) used computer models to determine that before China took control measures on 23 January, most COVID-19 patients had not been recorded. This was the main reason for the rapid spread of the epidemic [12].

One of the most important features of an epidemic is the spatial transmission, which mainly depends on epidemic mechanisms, human mobility, and control strategies [13]. When deciding on timetables for local reopening activities, the spatio-temporal characteristics and trends of COVID-19 need to be considered [14]. A timely understanding of the temporal and spatial patterns of COVID-19 transmission is the key to formulating epidemic prevention and control measures and allocating medical resources [15]. Moh et al. (2020) analyzed the temporal and spatial dynamics of the COVID-19 pandemic in Kuwait, finding that COVID-19 had significant patterns of transmission and aggregation among migrant workers, citizens, and residential communities [15]. Wang et al. (2020) proposed that comprehensively understanding the spatio-temporal dynamics and trends of COVID-19′s epidemic situation is very important for strengthening public health intervention and significantly reducing the incidence of the disease [14]. Sun et al. (2020) found that the COVID-19 epidemic in South Korea had spatial clustering characteristics, and identified 12 statistically significant clusters [16]. Chen et al. (2020) used a Bayesian spatio-temporal model to determine the distribution of COVID-19 cases in the early stage of the epidemic, and assessed its correlation with population migration in Wuhan [17]. Kang et al. (2020) analyzed the spatial transmission of COVID-19 in China, and found there were significant spatial correlations associated with COVID-19 infections starting from 22 January 2020 [18].

There are two deficiencies in current research on COVID-19: (1) As opposed to the national or provincial (state) level, there has been insufficient research on the small scale of cities, counties, and towns. However, the impact of the COVID-19 epidemic differs significantly across different regions and each country or region needs to take control measures according to the local situation. Therefore, identifying the rules governing the spread of the COVID-19 epidemic on a micro-scale can help the government develop accurate policies. (2) The current research has been mainly based on the number of confirmed cases and deaths, generally with a single research index. It is important to further explore detailed information about COVID-19 patients to reflect the spatio-temporal characteristics of COVID-19 transmission in the region from many perspectives. Based on that, by mining the information on 417 COVID-19 patients in Shenzhen, this study analyzed the characteristics of the epidemic transmission from multiple aspects in order to fill the gaps in the current research. The primary objective of this study was to further understand the characteristics and laws of COVID-19 spread in metropolises outside Wuhan, by conducting an in-depth analysis of the transmission process of COVID-19 in Shenzhen. Another goal was to provide a scientific reference for relevant government departments to predict the temporal and spatial evolutionary trends associated with the spread of COVID-19, and to formulate effective prevention and control policies.

Based on the case information of COVID-19 patients in Shenzhen, this study first analyzed the time series characteristics of the development of the epidemic. Then, the spatial evolution of COVID-19 was analyzed, using the method of spatial analysis. Then, based on the relevant literature and data availability, indicators were selected from three perspectives of the economy, society, and the environment. A multiple linear regression method was then used to explore the factors influencing COVID-19 transmission. Finally, the paper closes with a discussion of the analysis results.

## 2. Materials and Methods

### 2.1. Overview of the Research Area

After the COVID-19 outbreak broke out in Wuhan, China, the epidemic gradually spread to most parts of the country. Shenzhen was one of the most severely affected areas outside Hubei Province, which was typical. On 19 January 2020, the Shenzhen Municipal Health Commission (SMHC) reported the first confirmed case of COVID-19. By 12 August, a total of 462 cases of COVID-19 had been reported, including 423 domestic cases and 39 imported cases [19]. A total of 417 domestic cases were confirmed before 22 February, while the other 6 domestic cases were imported from other regions after 31 March. Therefore, January and February were the main periods of the epidemic in Shenzhen, making the epidemic data before 22 February representative.

**Figure 1:**
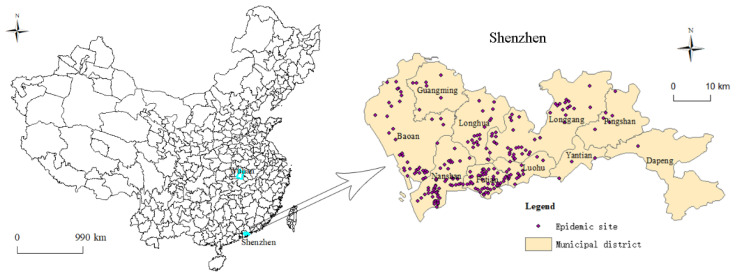
The study area and distribution of epidemic sites.

Shenzhen is a coastal city in the south of China. It is adjacent to Hong Kong and is approximately 1100 km away from Wuhan City (Figure 1). It is a national economic center and an international city. In recent years, Shenzhen’s GDP has always been among the highest in China, with an urbanization rate reaching 100%. Shenzhen has ten administrative districts, with a total area of 1997.47 km^2^. In 2019, the permanent resident population was 13,438,800 people.

### 2.2. Data and Variables

#### 2.2.1. Data Source

The data for this study mainly included COVID-19 case data and socio-economic data. The COVID-19 case data came from the SMHC and included two data segments. One segment included the details for the 417 domestic cases reported by the SMHC before 22 February. We sorted by the patient’s gender, age, date of symptom onset, date of hospitalization, travel history, and other information. The second data segment reported the places where COVID-19 patients stayed once the symptoms had onset, which was announced by the SMHC after 31 January. A total of 242 epidemic sites were reported by 22 February (Figure 1). The incubation period of COVID-19 is usually within 14 days [20]. Therefore, we selected three time nodes for analysis: 31 January, 14 February, and 22 February. Due to a lack of available socio-economic data for Shenzhen in 2020, statistical data for 2018 were used as an alternative for this study. Those data were mainly derived from “Shenzhen Statistical Yearbook 2019”.

#### 2.2.2. Variable Selection

The spread of COVID-19 was heterogeneous over space, and the epidemic’s development was affected by a variety of factors. For this study, eight indicators were selected from economic, social, and environmental perspectives to explore the factors influencing COVID-19 transmission. (1) From an economic perspective, GDP was used to represent the level of regional economic development. Areas with high economic level are usually more attractive for people and have more intensive economic activities. The actual utilization of foreign capital represented the degree of economic openness. The per capita disposable income index represented residential income. (2) From a social perspective, because the virus is carried and transmitted by human beings, the resident population and population density were selected to represent the current population situation in society. The regions with a large number of industrial enterprises tend to attract more migrant workers. The number of industrial enterprises represented the vitality of production and commercial activities. (3) In terms of the environment, the green coverage ratio represented the greening level of the area. The index assessing good air quality rate represented the air pollution status.

### 2.3. Research Methods

#### 2.3.1. Nearest Neighbor Index

The spatial distribution types of point elements are generally divided into three types: aggregate distribution, uniform distribution, and random distribution. In order to explore the spatial distribution types of epidemic sites in Shenzhen, we used the nearest neighbor index (NNI) method for measurement and analysis. The NNI is a common index used to judge the spatial distribution type of point elements [21]. The calculation formula of the NNI is as follows:

(1) $$\begin{array}{l} \mathrm{NNI}=\frac{\bar{P_{1}}}{\bar{P_{e}}}\\ \bar{P_{e}}=\frac{1}{2\sqrt{D}}=\frac{1}{2\sqrt{N/A}} \end{array}$$

In Equation (1), $\bar{P_{1}}$ is the measured mean distance between nearest neighbor point; $\bar{P_{e}}$ is expected mean distance if all points are randomly distributed; *D* is the point density; N is the total number of points; *A* is the area of the study area. If NNI < 1, it indicates that the epidemic sites are clustered. If NNI = 1, the points are randomly distributed. If NNI > 1, the points are uniformly distributed. This study used ArcGIS10.1 software to measure NNI of epidemic sites. The Z value in the result was used to determine whether to accept or reject the null hypothesis (the null hypothesis is that the distribution is random). A Z value greater than 1.65 or less than −1.65 indicated that the observed spatial pattern was unlikely to reflect a random pattern.

#### 2.3.2. Kernel Density Analysis

In order to study the spatial distribution density of epidemic sites, the kernel density estimation (KDE) method was used to measure and analyze this aspect. The KDE method takes the position of each sample point as the center, and calculates the density contribution value of each grid unit of each sample point in the specified range (circle with radius h) through the kernel density function. The closer the distance from the center of the sample point is, the greater the density is. The same method is used to calculate each sample point in the region, and the density at the same location is superimposed to obtain the kernel density of the points in the whole region [22]. Visualizing KDE results using ArcGIS software can better display the concentrated area of epidemic sites in space. The calculation formula of KDE is:

(2) $$f(s)=\sum_{i=1}^{n}\frac{1}{h^{2}}k\left(\frac{s-s_{i}}{h}\right)$$

In this expression, *f*(*s*) is the estimated kernel density at the space position s; $k\left(\frac{s-s_{i}}{h}\right)$ is a kernel function; $\left(s-s_{i}\right)$ is the distance between the estimated point s and the sample point $s_{i}$; *n* is the number of points within a certain range.

#### 2.3.3. Standard Deviational Ellipse

The standard deviational ellipse (SDE) method is used to analyze the spatial diffusion direction and the changing trend of the center of gravity of the epidemic site. The SDE is a method for studying the direction of distributions and the features of spatial point elements [23]. SDE can accurately reveal the center, dispersion, and direction trend of the spatial distribution of geographical elements. The long axis direction of the ellipse represents the direction with more distributed elements. The short axis is the opposite; the greater the difference between the long and short axes is, the stronger the directivity of the element. The formulas are calculated as follows:

Weighted average center:

(3) $${\bar{X}}_{\omega }=\frac{\sum_{i=1}^{n}\omega_{i}y_{i}}{\sum_{i=1}^{n}\omega_{i}},{\bar{Y}}_{\omega }=\frac{\sum_{i=1}^{n}\omega_{i}y_{i}}{\sum_{i=1}^{n}\omega_{i}}$$

Elliptic direction:

(4) $$\begin{array}{l} \tan\theta =\frac{A+B}{C}\\ A=\left(\sum_{i=1}^{n}\omega_{i}^{2}{\tilde{x_{i}}}^{2}-\sum_{i=1}^{n}\omega_{i}^{2}{\tilde{y_{i}}}^{2}\right)\\ B=\sqrt{{\left(\sum_{i=1}^{n}\omega_{i}^{2}{\tilde{x_{i}}}^{2}-\sum_{i=1}^{n}{\tilde{y_{i}}}^{2}\right)}^{2}+4\sum_{i=1}^{n}\omega_{i}^{2}{\tilde{x_{i}}}^{2}{\tilde{y_{i}}}^{2}}\\ C=2\sum_{i=1}^{n}\omega_{i}^{2}\tilde{x_{i}}\tilde{y_{i}} \end{array}$$

Standard deviation of the *X*-axis:

(5) $$\sigma_{x}=\sqrt{\frac{{\sum_{i=1}^{n}\left(\omega_{i}\tilde{x_{i}}\cos\theta -\omega_{i}\tilde{y_{i}}\sin\theta \right)}^{2}}{\sum_{i=1}^{n}\varpi_{i}^{2}}}$$

Standard deviation of the *Y*-axis:

(6) $$\sigma_{y}=\sqrt{\frac{{\sum_{i=1}^{n}\left(\omega_{i}\tilde{x_{i}}\sin\theta -\omega_{i}\tilde{y_{i}}\cos\theta \right)}^{2}}{\sum_{i=1}^{n}\varpi_{i}^{2}}}$$

In Equations (3)–(6), (*x_i_, y_i_*) is the spatial coordinate of the research object and $\omega_{i}$ represents the weight at the spatial element *i*. The (${\bar{X}}_{\omega }$,${\bar{Y}}_{\omega }$) represents the weighted average center of the spatial data set of the study object. The symbol *θ* is the azimuth angle of ellipse; $\tilde{x_{i}}$ and $\tilde{y_{i}}$ indicate the deviation of coordinates between the spatial coordinates of the object and the average center. The symbols $\sigma_{x}$ and $\sigma_{y}$ represent the standard deviation between the X axis and Y axis.

#### 2.3.4. Multiple Linear Regression

We used the multiple linear regression (MLR) method to explore which factors have influenced the spread of COVID-19. This study used SPSS 21.0 software (IBM, Armonk, NY, USA) to calculate MLR.

## 3. Results

### 3.1. Structural Features of Cases

**Figure 2:**
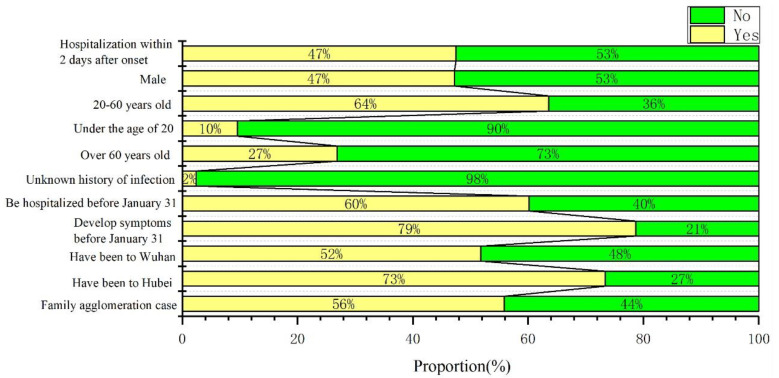
Case structure of COVID-19 in Shenzhen, China.

Figure 2 shows that the proportion of male patients with COVID-19 in Shenzhen was essentially the same as for females, and there were infected people in all age groups. The majority of patients were between 20 and 60 years old and the proportion of patients under 20 years old was relatively small. A total of 79% of the patients developed symptoms before 31 January. However, 60% of patients were hospitalized before 31 January. Approximately 47% of the patients were hospitalized within 2 days after the onset of the disease. In terms of travel history, 73% of the patients had traveled to Hubei province before being diagnosed. A total of 52% of the patients had been to Wuhan. Therefore, most of the patients who had been to Hubei had a history of traveling in Wuhan. Only 2% of patients had an unknown history of infection. Family agglomeration cases accounted for 56%, indicating that COVID-19 had spread widely within the family. This indicates that either Shenzhen did not experience community transmission of COVID-19, or it was controlled at the initial stage of community transmission.

### 3.2. Time Series Variation Characteristics

**Figure 3:**
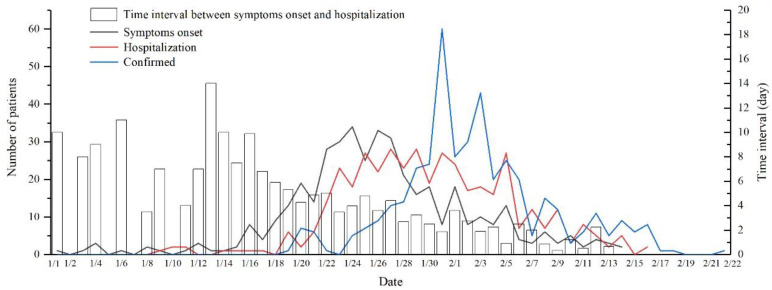
Temporal variation in COVID-19 cases.

Figure 3 shows three types of curves with different variation characteristics, as follows: (1) There was a period of slow growth from 19 January to 26 January. This was the early stage of the outbreak, with fewer than 10 new cases confirmed each day. (2) 27 January to 3 February was a period of rapid growth. The number of confirmed cases rose at this stage, peaking on 31 January, with an average daily increase of 29 confirmed cases. (3) 4 February to 22 February, there was a gradual decline. The number of new confirmed cases per day gradually decreased, and the epidemic had been effectively brought under control.

With respect to patient onset, 1 January was the earliest day that patients showed symptoms. This was 18 days before the official date of the first confirmed diagnosis. 14 February was the final date of onset, 8 days earlier than the date of confirmed diagnosis. The development of the epidemic showed the following characteristics with respect to the date when the patient first developed symptoms: (1) A germination stage occurred from 1 to 18 January, shown by the steady change in the curve and the small number of patients. (2) From 19 January to 5 February, there was a period of sharp growth. The number of cases increased significantly, reaching an average of 19 cases per day and peaking on 24 January. (3) From 6 February to 14 February, there was a recession period. The number of cases decreased, with less than 10 cases per day. The hospitalization curve and the symptoms onset curve have similar characteristics, but show significant hysteresis. Hospitalizations began on 9 January and all patients were hospitalized before 16 February. The daily number of hospitalized patients remained in single digits before 21 January, gradually increased after 22 January, and then declined after 6 February.

All 417 patients in Shenzhen showed hysteresis characteristics, from the onset of symptoms to hospitalization and to confirmed diagnosis. The time interval from the onset of symptoms to hospitalization showed significant phasic characteristics. Figure 3 shows that between 1 January and 27 January, there was a longer time interval from onset to patient hospitalization; after that, the time interval was significantly shorter. The average time interval between onset and hospitalization prior to 17 January was longer than 6 days, peaking on 13 January. There were few cases of hospitalization immediately after onset (i.e., the time interval from onset to hospitalization is 0 days) throughout the period.

### 3.3. Spatial Distribution Characteristics

#### 3.3.1. Nearest Neighbor Index Analysis

The Average Nearest Distance tool in ArcGIS10.1 was used to analyze the NNI of epidemic sites in Shenzhen on 31 January, 14 February, and 22 February. The results are shown in Table 1.

Table 1 shows that the NNI of epidemic sites at the three time nodes in Shenzhen City was less than 1, and Z values were all less than −1.65. This indicates that the epidemic sites had a concentrated distribution. Specifically, the NNI values were 0.80, 0.72, and 0.70 on 31 January, 14 February, and 22 February respectively, and were significant at the 1% level. The NNI value gradually decreased, indicating that the concentration of epidemic sites consistently increased over the study period. The NNI was 0.08 lower on 14 February compared to 31 January, and the NNI was 0.02 lower on 22 February compared to 14 February. This indicated that the epidemic sites were concentrating at a lower speed during this period.

#### 3.3.2. Kernel Density Analysis

**Figure 4:**
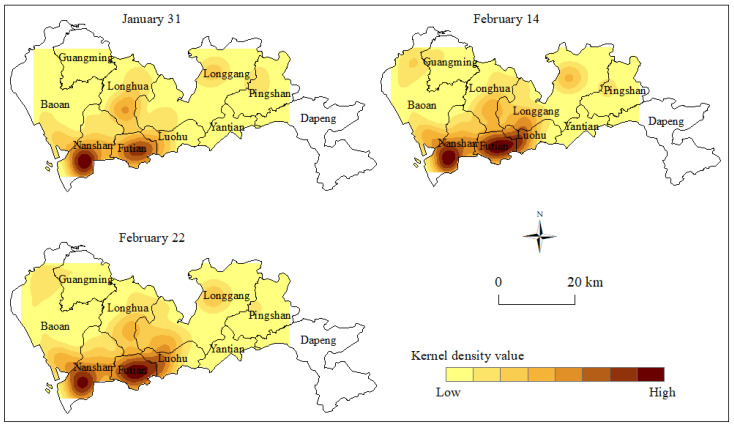
The kernel density of the spatial distribution of epidemic sites.

The kernel density analysis tool in ArcGIS software was used to generate a kernel density map of epidemic sites at the 3 time nodes (Figure 4). Figure 4 shows that the 3 time nodes had different characteristics of kernel density. 31 January was during the early stage of the epidemic disease outbreak, and a clear core area appeared in space. This represented two high-density agglomerations that formed in Nanshan District and Futian District, and two medium-density agglomerations that formed in Longhua District and Longgang District. This resulted in a multi-core distribution structure in space. As the epidemic developed further, the core area expanded significantly on 14 February, and a new core area was derived. The two high-density agglomeration areas in Nanshan District and Futian District showed a near-regional expansion trend between 31 January and 14 February. At the same time, the medium-density agglomeration area of Longhua District also further expanded, forming a continuous area with the high-density agglomeration area. In addition, Baoan District added a medium-density agglomeration area.

At the end of the epidemic stage on 22 February, the core area of the epidemic site had stabilized, with some areas showing a small expansion. At the end of the epidemic development, the daily number of new epidemic sites had gradually decreased. The high-density core areas located in Nanshan District and Futian District had changed little. The medium-density core areas of Baoan District and Longgang District also maintained their original characteristics. The medium-density core area of Longhua district and Guangming District showed a small expansion trend.

#### 3.3.3. Standard Deviational Ellipse Analysis

**Figure 5:**
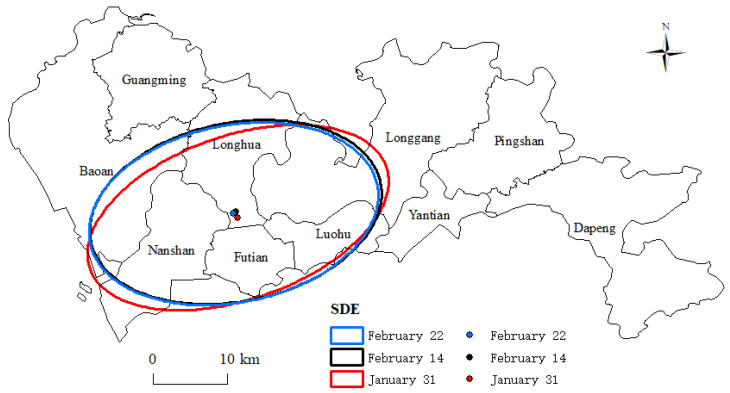
The SDE of the spatial distribution of epidemic sites.

When further analyzing the direction of the spread of the epidemic site, the SDE analysis results showed significant differences in the long and short axes of SDE at different time nodes. This indicated that the spread of epidemic sites in Shenzhen had a clear direction. Figure 5 shows that the principal axis of the SDE was consistently along the “northwest–southeast” direction of the city. The spreading angles of the epidemic sites on 31 January, 14 February, and 22 February were 70.9°, 78.5°, and 78.6° (Table 2), respectively. The center of gravity of the ellipse was consistently in the southern part of Longhua District; it first moved to the northwest and then shifted to the southwest.

### 3.4. Analysis of Factors Influencing the Spread of COVID-19

The number of confirmed cases in 10 administrative areas of Shenzhen city was taken as the dependent variable and the economic, social, and environmental indicators as the independent variables to conduct multiple linear regression analysis. Table 3 shows that the variance inflation factor (VIF) values in the regression results were all less than 10, indicating the problem of multicollinearity between independent variables can be ignored. The adjusted R^2^ was 0.985, indicating that the model had a good degree of fit.

With the exception of the green coverage rate and good air quality rate, other indicators were significant within a 95% confidence interval. This indicated that economic and social factors impacted the spread of the epidemic. Specifically, the regression coefficients of GDP, actual utilization of foreign capital, resident population, population density, and the number of industrial enterprises were positive. This indicated the variables were associated with an increase in the spread of COVID-19. Of these, the regression coefficient value of the number of industrial enterprises was the largest; that is, it had the greatest impact on the development of the epidemic. The regression coefficient of the per capita disposable income was negative, indicating that this factor had a negative effect on the spread of COVID-19. The regression coefficients of the two environmental indicators were negative, but did not pass the significance test. This indicated environmental factors had no significant effect on the spread of the epidemic. Overall, social factors had a relatively large impact on the epidemic.

## 4. Discussion

To date, most studies on the spatio-temporal characteristics of COVID-19 transmission have been based on the analysis of the changes in the number of confirmed cases or deaths. This study analyzed the evolutionary characteristics of the epidemic situation from five aspects: the time of onset, hospitalization, case confirmation, the time interval from onset to hospitalization of COVID-19 patients, and the epidemic site. This approach clearly revealed the laws by which the epidemic spread within the city.

The analysis of the case structure of 417 patients showed that the natural structure of the population (age, gender, etc.) showed no significant difference in the infection of the virus. Human behavior was likely the driver for virus transmission, based on four key facts. (1) In the early stage, due to insufficient awareness, the government lacked effective control measures to prevent and control the spread of the virus. In particular, it did not attract public attention through outreach. Even in Wuhan, people were not taking protective measures, such as wearing masks [11]. (2) The timing of the virus coincided with the Spring Festival, which is the most mobile and largest festival in China. During this time, people gather more frequently, and activities are more intensive, significantly increasing the probability of being infected with the virus [7]. (3) People with a history of travel in epidemic areas did not take medical isolation measures in time, increasing the risk of transmission. Wuhan City, Hubei Province, adopted a city closure measure on 23 January, and Shenzhen did not adopt quarantine measures for people with a history of traveling in Hubei until after that. During this lag in intervention, patients may have infected others before being quarantined. In addition, patients did not receive treatment in time after the onset of the disease, increasing the exposure time. (4) Among the 417 patients in Shenzhen, 55.9% were likely infected as a result of family agglomeration. Close contact between friends and relatives can lead to a relaxation of vigilance, leading to infection.

Patients usually show COVID-19 symptoms before diagnosis. Therefore, the initial onset time can effectively reflect the spread of COVID-19. The number of people with symptoms was relatively small from 1 January to 18 January, and increased sharply from 19 January to 5 February. This may have been related to Chinese Spring Festival transportation. 10 January is the first day of China’s Spring Festival transportation, and the population generally begins to move on a large scale then. The early confirmed cases in Shenzhen were, therefore, mainly imported from Hubei Province. The migration brought about by the Spring Festival led to a small number of symptomatic patients in Shenzhen before 18 January. However, before 18 January, Shenzhen had no confirmed cases and no strong interventions, except to recommend wearing masks and avoiding visits to densely populated areas. These were not mandatory requirements. In the days around the Chinese New Year (25 January), passenger traffic peaked, leading to a wider spread of COVID-19 among the population. As a result, there was a sharp increase in the number of people who developed symptoms after 19 January. After 7 February, the Shenzhen government placed all residential areas under closed management. This stipulated that no outsiders or vehicles were allowed to enter the community, no residents were allowed to visit or dine together, and there was strict implementation of a mask requirement in public places. The implementation of strong control measures has effectively controlled the spread of COVID-19 in Shenzhen, leading to a decline in the number of cases since 6 February.

After 27 January, there was a significant shortening in the interval from onset to patient hospitalization. As the epidemic has developed, the public has developed a deeper understanding of COVID-19 and seeks timely treatment. However, the timing is also closely related to national policies. On 22 January, the National Ministry of Finance and the Healthcare Security Administration jointly issued a notice informing COVID-19 patients that the financial department would subsidize their personal expenses [24]. Such policies ensure that patients do not avoid medical care due to cost problems, so patients can isolate and receive treatment more rapidly, reducing transmission. There was a significant increase in the number of patients hospitalized after 22 January. This also demonstrates that national policies have significantly impacted COVID-19 patients.

Most studies to date on the spatial distribution patterns of COVID-19 have adopted the spatial autocorrelation method [16,18]. However, the number of confirmed cases, the number of deaths, and other indicators around the world have been expressed at a county or city scale, which cannot effectively reflect the spatial evolution characteristics within a city. As such, we analyzed the spatial evolution characteristics of epidemic sites by using NNI, kernel density, and SDE methods, providing a useful supplemental approach for studying the spatial distribution pattern of COVID-19. The analysis results show that the epidemic sites were spatially clustered; some studies have also found spatial clustering of COVID-19 at the national scale [14,16,25]. In Shenzhen, the epidemic sites have been mainly located in Futian District, Luohu District, and Nanshan District. These three districts rank high with respect to population density and per capita GDP, and all have railway stations within their districts (Shenzhen has five railway stations in total).

A recent study showed that railway carriages were associated with the COVID-19 spread and regional outbreak in China [26]. Using a multiple linear regression analysis, we found that economic and social indicators significantly impacted COVID-19 transmission. This is consistent with Matthew et al., who found that heterogeneity of COVID-19 in New York City in the United States was largely driven by neighborhood socio-economic conditions [27]. Person-to-person contact increases the risk of COVID-19 infection [2,6,28]. Once COVID-19 is transmitted in an area, agglomeration is more likely to occur in densely populated areas [16]. Environmental factors did not show significant effects on the spread of COVID-19, which was consistent with Mollalo et al.’s findings [29], however, some studies have found different results [9,30].

Shenzhen has controlled the epidemic through a series of measures, gradually restoring orderly production and life. However, COVID-19 continues to spread around the world, and since July, there has been a COVID-19 outbreak in Hong Kong, which is adjacent to Shenzhen. As an international metropolis, Shenzhen should continue to adhere to the strategy of preventing internal proliferation, preventing the import of the virus, and placing the highest priority on epidemic prevention and control.

Like all studies, this research had limitations. We analyzed the temporal changes of the onset, hospitalization, and diagnosis of COVID-19 patients; however, patients showed considerable transmission potential before the onset of symptoms [31]. Therefore, the transmission of COVID-19 in Shenzhen may have occurred earlier than the onset of symptoms. Other epidemiological variables and clinical characteristics, such as latency, were not collected, and there was a lack of research on the premorbid condition of patients. In addition, the factors affecting the spread of the virus are complex, and involve both quantifiable and non-quantifiable indicators. In this paper, the evaluation index system of COVID-19 transmission factors was constructed based on data accessibility, which means it did not consider other indicators that were difficult to quantify. Scientifically selecting evaluation indexes is important for future studies.

## 5. Conclusions

This study examined 417 COVID-19 patients in Shenzhen, China. Spatial analysis and multiple linear regression methods were used to explore the spatial and temporal characteristics and influencing factors of COVID-19 transmission in Shenzhen. Four key conclusions were reached: (1) From the perspective of temporal variation, COVID-19 transmission in Shenzhen went through three stages: the germination stage, the rapid growth stage, and the decline stage. (2) From the perspective of spatial distribution, COVID-19 transmission was characterized by significant spatial clustering, and the degree of clustering consistently increased. COVID-19 transmission was directional, forming a “core area” in space. (3) Economic and social factors drove the spread of COVID-19, while environmental factors did not have a significant impact on the epidemic. (4) With respect to the spread of COVID-19, the prevention and control measures taken by the Shenzhen government at different times determined the developmental trend of the epidemic. This study reveals the process and characteristics of COVID-19 transmission in metropolises outside Wuhan, providing a reference for epidemic prevention and control in other cities around the world.

## Figures and Tables

**Table 1:** NNI (nearest neighbor index) of epidemic site distribution.

Date	Sample Number	NNI	Z Value	Significance Level
31 January	82	0.80	−3.46	1%
14 February	230	0.72	−8.03	1%
22 February	242	0.70	−8.79	1%

**Table 2:** The parameters of the SDE at different time nodes.

Time	Rotation	X-StdDist	Y-StdDist
31 January	70.9°	0.09	0.19
14 February	78.5°	0.10	0.18
22 February	78.6°	0.10	0.18

**Table 3:** Multiple linear regression results.

	Coefficient	95% CI	*p*-Value	VIF
GDP	0.102	(0.000, 0.000)	0.007	6.260
Actual utilization of foreign capital	0.092	(−0.003, 0.003)	0.004	7.002
Per capita disposable income	−0.018	(−0.005, 0.004)	0.013	4.128
Resident population	0.137	(−0.051, 0.042)	0.029	4.068
Population density	0.479	(−0.010, 0.010)	0.008	5.823
Number of industrial enterprises	1.397	(−0.050, 0.050)	0.018	8.152
Green coverage rate	−0.187	(−4.403, 2.822)	0.652	2.655
Good air quality rate	−0.373	(−12.122, 9.503)	0.389	3.496
Adjusted R^2^	0.985			
Significance F	0.012			
